# Artificial intelligence-driven imaging-based prediction models for hemorrhagic transformation after mechanical thrombectomy in acute ischemic stroke: a systematic review and meta-analysis

**DOI:** 10.3389/fneur.2026.1960029

**Published:** 2026-10-20

**Authors:** Pan Hao, Sen Wang, Ruiqiang Xin

**Affiliations:** Beijing Luhe Hospital, Capital Medical University, Beijing, China

**Keywords:** acute ischemic stroke, artificial intelligence, hemorrhagic transformation, mechanical thrombectomy, medical imaging

## Abstract

**Objective:**

Hemorrhagic transformation (HT) is a major complication after mechanical thrombectomy (MT) in acute ischemic stroke (AIS). Although artificial intelligence (AI)-driven imaging-based prediction models have shown promise for predicting HT, their overall predictive performance remains uncertain. This systematic review and meta-analysis evaluated their predictive performance.

**Methods:**

PubMed, Embase, Cochrane Library, Web of Science, CNKI, Wanfang, VIP, and CBM/SinoMed were searched from inception to July 2, 2026. Studies developing or validating AI-driven imaging-based prediction models for predicting HT after MT in AIS, including both pre- and post-procedural imaging predictors, were included. A random-effects model was used to pool the area under the curve (AUC), while a bivariate random-effects model was used to jointly estimate pooled sensitivity and specificity and construct the SROC curve. Subgroup analyses explored potential sources of heterogeneity.

**Results:**

Eleven studies (total *N* = 3,541) were included, 10 studies contributed eligible AUC estimates, with evaluation cohorts ranging from 18 to 261 participants. The pooled AUC was 0.891 (95% CI: 0.853–0.928), with low between-study heterogeneity (*I*^2^ = 29.0%, *p* = 0.238). The bivariate random-effects model yielded a pooled sensitivity of 0.666 (95% CI: 0.578–0.744) and a pooled specificity of 0.919 (95% CI: 0.842–0.960). Exploratory subgroup analyses did not provide robust evidence supporting the superiority of any specific subgroup, and their interpretation was limited by small subgroup sizes and methodological heterogeneity. However, these findings should be interpreted cautiously because of the limited number of studies within individual subgroups.

**Conclusion:**

AI-driven imaging-based prediction models showed generally favorable discrimination in predicting HT after MT in AIS. However, current evidence does not establish the superiority of any specific model type. The predominance of retrospective studies, limited external validation, and methodological heterogeneity limit the generalizability of current findings. Large, multicenter prospective studies with rigorous external validation are needed before clinical implementation.

**Systematic review registration:**

The systematic review was registered with PROSPERO (CRD420261441463).

## 1 Introduction

Acute ischemic stroke (AIS) remains one of the leading causes of death and long-term disability worldwide, imposing a substantial socioeconomic burden on healthcare systems (1). Over the past decade, mechanical thrombectomy (MT) has substantially improved the treatment of AIS caused by large vessel occlusion, with multiple randomized controlled trials demonstrating its efficacy in improving functional outcomes (2). Current international guidelines strongly recommend MT as the standard of care for eligible patients with anterior circulation large vessel occlusion within 24 h of symptom onset.

Despite the established efficacy of MT in restoring cerebral blood flow, hemorrhagic transformation (HT) remains one of the most serious complications after this procedure. HT encompasses a spectrum ranging from asymptomatic hemorrhagic infarction to symptomatic parenchymal hematoma, and occurs in approximately 30–50% of patients following MT, with hemorrhagic events associated with worse functional outcomes (3). Symptomatic intracranial hemorrhage (sICH), the most severe form of HT, has been reported in 5%–10% of patients and is strongly associated with poor functional outcomes and increased mortality (4). The pathophysiology of HT is complex and multifactorial, involving blood–brain barrier disruption, reperfusion injury, endothelial dysfunction, and post-recanalization inflammatory cascades (5). Given the limited therapeutic options once HT occurs, early and accurate prediction of HT risk may facilitate risk stratification and closer neurological monitoring during the periprocedural period (6).

Traditional methods for predicting HT after MT have primarily relied on clinical risk scores (e.g., TAG, ASIAN, and SEDAN scores), individual clinical or imaging markers (e.g., ASPECTS and admission glucose), or conventional logistic regression models (7, 8). However, the predictive performance of these approaches is often modest, particularly when evaluated in external validation cohorts (9). Conventional approaches may also have limited capacity to capture complex nonlinear interactions among multiple predictors, which may constrain predictive performance (10).

In recent years, the rapid advancement of artificial intelligence (AI)—particularly the widespread application of machine learning (ML) and deep learning (DL) algorithms in medical image analysis—has opened new avenues for improving HT prediction (11, 12). AI techniques enable automatic extraction of high-dimensional quantitative features from medical images and facilitate nonlinear pattern recognition, potentially reducing reliance on subjective visual interpretation. Concurrently, medical imaging plays an integral role in the assessment of AIS patients undergoing MT. Different imaging modalities and techniques—including diffusion-weighted imaging (DWI), perfusion-weighted imaging (PWI), CT perfusion (CTP), CT angiography (CTA), and digital subtraction angiography (DSA)—provide complementary information on ischemic tissue, collateral circulation, and cerebral hemodynamics (13). Imaging acquired at different stages of the MT pathway may provide distinct information relevant to HT risk. The integration of AI algorithms with imaging data holds promises for capturing subtle imaging patterns associated with HT and improving risk prediction (14).

Although several recent reviews have summarized AI-based HT prediction in stroke (15–18), the present study specifically focused on AIS patients undergoing MT, with particular attention to peri-procedural imaging-based prediction models. To fill this gap, the present study provides a systematic review and meta-analysis specifically restricted to AIS patients undergoing MT, with a particular focus on peri-procedural imaging predictors and a quantitative comparison of image-only versus image-plus-clinical models. The included studies employed various AI approaches, including machine learning, deep learning, and radiomics-based models (14, 19, 20). These studies have reported encouraging predictive performance, with some models achieving AUC values exceeding 0.90 (21). However, considerable heterogeneity exists across studies in terms of imaging modalities, AI algorithms, sample sizes, validation strategies, and outcome definitions, which limits the comparability and generalizability of their findings. Therefore, a systematic synthesis of the available evidence is of substantial clinical and academic value, specifically to: (1) determine the pooled predictive performance of these models; (2) compare performance across imaging timing, predictor type, validation type, outcome definition, and country; (3) explore potential sources of heterogeneity across studies; (4) identify methodological gaps relevant to future model development and validation.

Against this background, the present study aimed to systematically evaluate, through a systematic review and meta-analysis, the predictive performance of AI-driven imaging-based prediction models for HT after MT in AIS patients, and to explore potential sources of heterogeneity through subgroup analyses stratified by imaging timing, predictor type, validation type, outcome definition and country. The findings of this study are expected to help clarify the current evidence on AI-based HT prediction and to inform future model development and validation.

## 2 Methods

### 2.1 Inclusion and exclusion criteria

#### 2.1.1 Inclusion criteria

This study was conducted in accordance with the Preferred Reporting Items for Systematic Reviews and Meta-Analyses (PRISMA) guidelines (22). The inclusion criteria were defined as follows: (1) patients with imaging-confirmed AIS who underwent MT (including studies referring to MT as endovascular therapy [EVT]); (2) prediction models based on AI methods, including but not limited to conventional ML (e.g., support vector machines, random forest, XGBoost), DL (e.g., convolutional neural networks, Transformer), and radiomics; (3) prediction models incorporating medical imaging or imaging-derived features, either alone or in combination with clinical or procedural variables, including but not limited to CT, CTA, CTP, MRI, and DSA; (4) outcome measures defined as HT-related intracranial hemorrhagic events in the original studies, including radiological HT or intracranial hemorrhage (ICH), sICH, and parenchymal hematoma type 2 (PH2), according to the definitions used in the original studies; (5) original prediction model studies reporting at least one extractable measure of predictive performance, such as AUC, sensitivity, or specificity.

#### 2.1.2 Exclusion criteria

Studies were excluded if they met any of the following criteria: (1) studies involving non-MT/EVT populations for which data from patients undergoing MT/EVT could not be separately extracted; (2) prediction models based solely on conventional statistical approaches, such as logistic regression or nomograms, without AI/ML methods or radiomics-based feature extraction/modeling; (3) prediction models without medical imaging or imaging-derived predictors; (4) outcomes unrelated to HT or intracranial hemorrhagic transformation, such as functional prognosis only; (5) inappropriate study types, including reviews, editorials, conference abstracts, case reports, animal studies, and studies without original data; and (6) duplicate publications or studies with substantially overlapping cohorts; when overlap was identified, the report providing the most complete and relevant data for the prespecified outcome and model evaluation was retained to avoid double counting.

### 2.2 Search strategy

A systematic literature search was conducted in the following databases from inception to July 2, 2026: PubMed, Embase, Cochrane Library, Web of Science, China National Knowledge Infrastructure (CNKI), Wanfang Data, VIP Chinese Science and Technology Periodical Database (VIP), and Chinese Biomedical Literature Database (CBM/SinoMed). A combination of MeSH terms and free-text words was used for the literature search. The core search terms encompassed: AIS-related terms (“acute ischemic stroke,” “cerebral infarction”), MT/EVT-related terms (“mechanical thrombectomy,” “endovascular treatment”), HT-related terms (“hemorrhagic transformation,” “intracranial hemorrhage”), and AI-related terms (“artificial intelligence,” “machine learning,” “deep learning,” “radiomics”). No restriction was placed on imaging modality in the search; studies using any imaging technique (e.g., NCCT, CTA, CTP, MRI, DSA) were considered eligible. The full search strategies for all databases are provided in the Supplementary materials. In addition, the reference lists of the included studies were manually screened to identify additional potentially eligible studies.

### 2.3 Study selection and data extraction

EndNote software was used to manage the retrieved records and to remove duplicates. Two reviewers independently screened the literature against the predefined inclusion and exclusion criteria. In the first step, titles and abstracts were reviewed to exclude obviously irrelevant records. Subsequently, the full texts of the remaining records were assessed for eligibility. Disagreements between the two reviewers were resolved through discussion; if consensus could not be reached, a third senior reviewer was consulted for the final decision.

A standardized data extraction form was used to independently extract study information. The extracted data covered six categories: (1) basic study information: first author, publication year, country, and study design; (2) participant characteristics: total sample size, sample size and number of HT events in the cohort used for model evaluation, and overall HT incidence; (3) imaging protocols: imaging modality, specific sequences or parameters, and imaging timing relative to MT (e.g., pre-procedural or peri−/post-procedural); (4) AI prediction models: model type (e.g., SVM, CNN, XGBoost, radiomics), predictor composition, training/validation strategy, and availability of independent external validation cohorts; (5) predictive performance metrics: AUC with corresponding 95% CI, sensitivity, specificity, calibration measures, and decision-curve analysis when available. Calibration and decision-curve data were extracted when available and were summarized descriptively because of heterogeneity in the reported methods and metrics; and (6) outcome definitions: criteria for HT diagnosis (e.g., ECASS criteria, Heidelberg Bleeding Classification [HBC]), and timing of outcome assessment.

For data extraction, priority was given to performance estimates from an independent external validation cohort. When external validation was unavailable, estimates from a prespecified internal validation or test set were extracted. If multiple eligible models were reported within the same cohort, the model evaluated using the most rigorous validation strategy was selected for the primary analysis; when validation strategies were equivalent, the model designated as the primary or final model by the original study was used. Sensitivity and specificity were extracted only when they corresponded to the same selected model, outcome definition, and evaluation cohort as the performance estimate used in the primary analysis. For studies that did not report the 95% CI of the AUC, the CI was estimated using the Hanley-McNeil method (23) based on the reported AUC and the numbers of patients with and without HT in the corresponding evaluation cohort. Potential cohort overlap was assessed based on study center, recruitment period, authorship, and the specific evaluation cohort from which the extracted performance estimate was derived. When broader source cohorts overlapped but the evaluation cohorts contributing to the meta-analysis were clearly non-overlapping, both studies were retained.

### 2.4 Quality assessment

The risk of bias of the included studies was assessed using the Prediction model Risk of Bias Assessment Tool (PROBAST+AI) (24, 25). The evaluation covered four domains: participants and data sources, predictors, outcomes, and analysis. Each domain was rated as “low risk,” “high risk,” or “unclear” according to the signaling questions and guidance provided by PROBAST+AI. Particular attention was paid to sample size relative to model complexity, model selection and validation procedures, calibration reporting, and potential overlap between predictors and outcome definitions. The assessments were performed independently by two reviewers, and disagreements were resolved through discussion.

### 2.5 Statistical analysis

Meta-analyses were performed using R software (R Foundation for Statistical Computing, Vienna, Austria), primarily with the meta, metafor, and mada packages. Given the anticipated heterogeneity across studies in terms of prediction models, imaging modalities, and outcome definitions, a random-effects model (26) was used to pool the reported AUC values with their 95% CIs. For studies reporting sufficient sensitivity and specificity data, a bivariate random-effects model was used to jointly estimate pooled sensitivity and specificity and to construct a summary receiver operating characteristic (SROC) curve. Heterogeneity in the pooled AUC analysis was assessed using the Cochran Q test and the *I*^2^ statistic, with *I*^2^ > 50% indicating substantial heterogeneity (27). Threshold effects were assessed using Spearman rank correlation analysis between logit-transformed sensitivity and the logit-transformed false-positive rate (1 − specificity).

To explore potential sources of heterogeneity, five exploratory subgroup analyses were conducted: (1) by imaging timing (pre-procedural vs. post-procedural); (2) by predictor type (image-only vs. image plus clinical); (3) by validation type (external vs. internal); (4) by outcome definition (e.g., radiological HT/ICH vs. sICH); (5) by country (China vs. non-China). Differences between subgroups were assessed using tests for subgroup differences. Owing to the limited number of included studies, meta-regression and formal assessment of publication bias were not performed.

Leave-one-out sensitivity analyses were performed for both the pooled AUC analysis and the bivariate random-effects model. For the pooled AUC analysis, each study was sequentially excluded and the pooled AUC was recalculated to assess the influence of individual studies on the overall estimate. For the bivariate analysis, each eligible study was sequentially excluded to assess the robustness of the pooled sensitivity and specificity estimates. A two-sided *p* < 0.05 was considered statistically significant.

### 2.6 Deviations from the registered protocol

Several deviations from the registered protocol occurred during the review process. The originally planned subgroup analyses by imaging technology and model type were not retained in the final analysis, whereas subgroup analyses by outcome definition and country were added during peer review and were therefore considered exploratory rather than prespecified. In addition, the eligibility criteria were broadened from AI-driven multimodal imaging models requiring at least two imaging sequences or parameters to imaging-based AI prediction models incorporating medical imaging or imaging-derived features more generally. These deviations are reported here for transparency, and the PROSPERO record has been amended accordingly.

## 3 Results

### 3.1 Study selection results

A total of 982 records were initially identified through database searching. After stepwise screening, 11 studies (14, 19–21, 28–34) involving 3,541 patients were finally included. The study selection process is illustrated in Figure 1.

**Figure 1:**
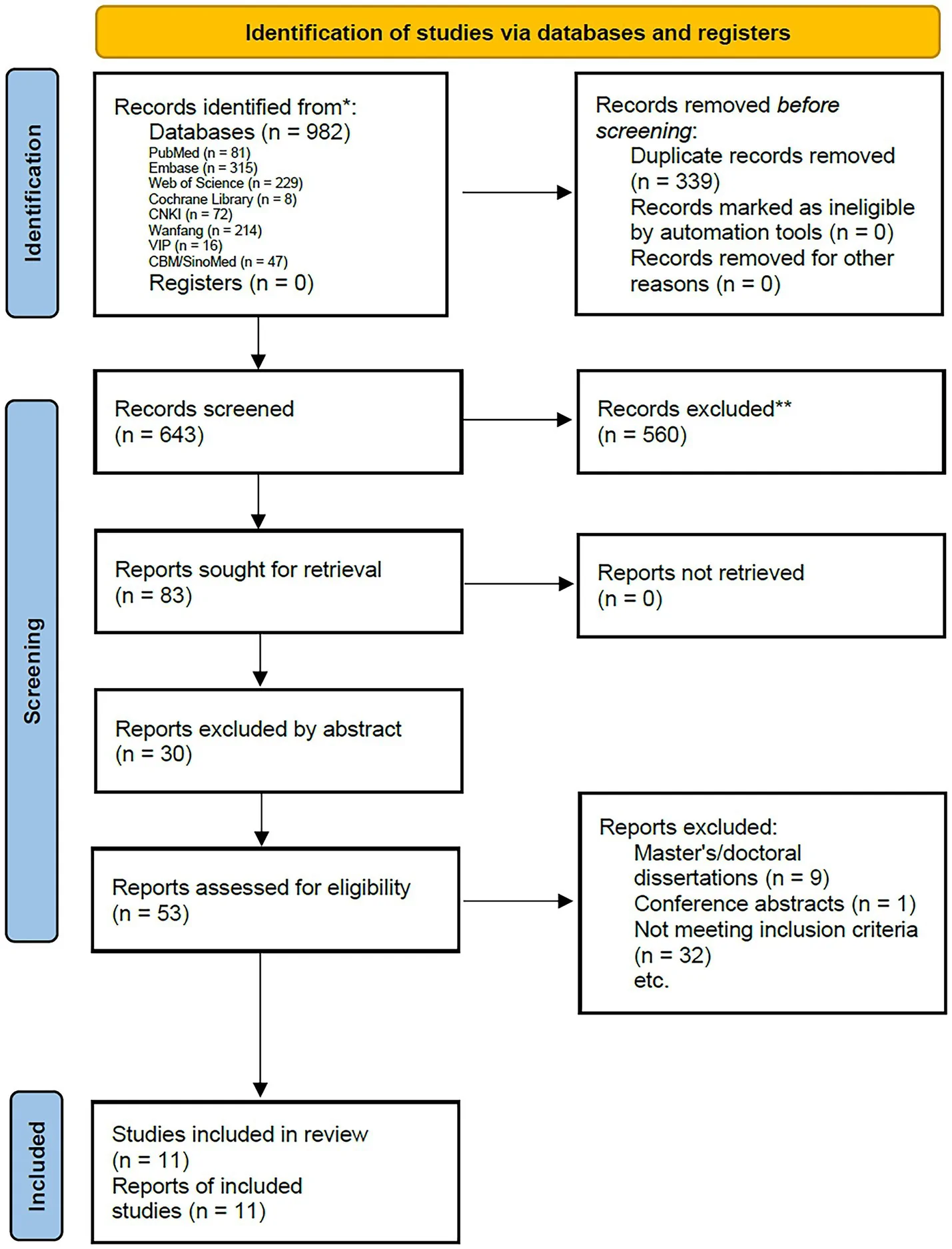
PRISMA 2020 flow diagram for study selection (22). CC BY 4.0. Source: PRISMA 2020 flow diagram template.

### 3.2 Baseline characteristics of the included studies

The 11 included studies were published between 2023 and 2026. All were cohort studies, with the majority being retrospective. Among them, four were multicenter studies, and three had independent external validation. The included studies were conducted in China, the United States, Korea, and Brazil, including both China and non-China cohorts. Sample sizes ranged from 105 to 922, with a median of 288. The proportions of the study-specific hemorrhagic outcomes ranged from 7.7 to 70.1%, with a median of 39.8%. The baseline characteristics of the included studies are summarized in Table 1.

**Table 1:** Study characteristics.

Study	Country	Design	Centers	Total N	Outcome events in the overall cohort, n (%)	Imaging modality	Imaging timing	Predictor type	Selected model	Validation	Evaluation cohort, n	Evaluation Events,n	Evaluation Non- events,n	AUC (95% CI)	Sensitivity/specificity	Outcome definition
Jiang et al. (14) 2023	China	Retrospective	2	392	103 (26.3)	Multiparametric MRI	Pre-procedural	Image + clinical	CNN	External	54	15	39	0.939 (0.852–1.000)*	0.859/0.890	HT
Wen et al. (19) 2023	China	Retrospective	1	105	52 (49.5)	NCCT	Pre-procedural	Image-only	Radiomics + LASSO + LR	Internal	32	16	16	0.797 (0.642–0.951)	0.625/0.875	HT
Heo et al. (32) 2023	Republic of Korea	Retrospective	1	202	109 (54.0)	DECT	Post-procedural	Image-only	3D CNN	Internal	23	16	7	0.911 (0.774–1.000)	0.688/0.857	HT
Li et al. (28) 2024	China	Retrospective	3	336	128 (38.1)	CTA	Pre-procedural	Image-only	Radiomics + LASSO + LR	External	106	49	57	0.850 (0.777–0.924)	0.592/0.982	ICH
Gong et al. (33) 2024	China	Retrospective	3	118	71 (60.2)†	NCCT	Pre-procedural	Image-only	Radiomics + LASSO + multivariable LR	Internal	36	22	14	0.877 (0.753–0.960)	0.773/0.786	HT
Jiang et al. (29) 2025	China	Retrospective + prospective temporal test	1	288	202 (70.1)	NCCT	Post-procedural	Image + clinical	Radiomics-DL hybrid model	Internal temporal	49	35	14	0.887 (0.783–0.990)	0.600/0.857	HT
Hu et al. (21) 2025	China	Retrospective	1	159	100 (62.9)	NCCT	Post-procedural	Image + clinical	Radiomics + SVM	Internal	32	20	12	0.925 (0.833–1.000)	0.900/0.833	HT
Hoffman et al. (20) 2025	USA	Retrospective	1	497	50 (10.1)	NCCT + CTA	Pre-procedural	Image + clinical	XGBoost	Internal	100	11	89	0.790 (0.670–0.890)	NR / NR	sICH
Goulart et al. (34) 2026	Brazil	Retrospective	1	351	27 (7.7)‡	CT	Pre-procedural	Image + clinical	XGBoost	Internal	69	5	64	NR	0.400/1.000	sICH
Li et al. (31) 2026	China	Retrospective	1	171	68 (39.8)	qDSA	Post-procedural	Image + clinical	ElasticNet + LR	Internal	18	7	11	0.860 (0.665–1.000)*	0.880/0.620	Broad HT
Li et al. (30) 2026	China	Retrospective	2	922	81 (8.8)	DSA + NCCT	Post-procedural	Image + clinical	TabPFN Transformer	External	261	20	241	0.955 (0.891–1.000)*	0.750/0.950	sICH

HT, hemorrhagic transformation; ICH, intracranial hemorrhage; sICH, symptomatic intracranial hemorrhage; NCCT, non-contrast computed tomography; CTA, computed tomography angiography; DECT, dual-energy computed tomography; DSA, digital subtraction angiography; qDSA, quantitative digital subtraction angiography; CNN, convolutional neural network; DL, deep learning; LASSO, least absolute shrinkage and selection operator; SVM, support vector machine; LR, logistic regression; TabPFN, Tabular Prior-Data Fitted Network; NR, not reported. Outcome event counts and percentages refer to the overall study cohort, whereas the “Evaluation cohort, n” column refers to the cohort used to obtain the performance estimate selected for the primary analysis. *The 95% CI was estimated from available data when it was not reported in the original study; upper confidence limits exceeding 1.000 were truncated at 1.000. ^¤^For Jiang et al. (14), the original study reported a sensitivity of 0.859 and a specificity of 0.890 in an external validation cohort of 54 patients (15 with HT and 39 without HT). These reported values could not be reconciled with a unique integer 2 × 2 contingency table. Therefore, the reported AUC was retained in the AUC meta-analysis, whereas this study was excluded from the bivariate sensitivity/specificity meta-analysis. ^†^Gong et al. (33) reported 71 HT events in the overall cohort, although subgroup counts reported elsewhere in the article were not fully consistent. ^¶^Jiang et al. (29) and Hu et al. (21) were conducted at the same institution and had overlapping source recruitment periods. However, the performance estimates included in this meta-analysis were derived from non-overlapping evaluation cohorts: the 32-patient test cohort in Hu et al. (21) was drawn from patients treated between June 2016 and November 2021, whereas the estimate from Jiang et al. (29) was obtained from a separate prospective temporal test cohort recruited between January and September 2023 (*n* = 49). ^‡^For Goulart et al. (34), the reported sensitivity and specificity corresponded to the XGBoost model for predicting sICH; the AUC for this selected model was not reported. Therefore, 27 sICH events were presented. ^§^Li et al. (31) used repeated 9:1 train-test splits across multiple random seeds; the reported AUC represents the mean test performance rather than the performance from a single fixed 18-patient test cohort. Because these performance estimates were averaged across repeated splits and did not correspond to a single reconstructable 2 × 2 table, Li et al. (31) was not included in the bivariate meta-analysis. Broad HT refers to the composite hemorrhagic outcome used in Li et al. (31), which included intracranial hemorrhagic findings and contrast extravasation according to the original study definition.

#### 3.2.1 Overall predictive performance

Ten studies reporting eligible AUC data were included in the pooled AUC analysis. Using a random-effects model, the pooled AUC was 0.891 (95% CI: 0.853–0.928) (Figure 2), with low between-study heterogeneity (*I*^2^ = 29.0%, *p* = 0.238). Eight studies reporting eligible paired sensitivity and specificity data were included in the bivariate analysis. Hoffman et al. did not report sensitivity and specificity, while Li H et al. reported averaged performance across repeated random train-test splits and therefore did not provide a single reconstructable 2 × 2 table for the bivariate analysis. For Jiang et al. (14), the reported sensitivity and specificity could not be reconciled with a unique integer 2 × 2 table for the stated evaluation cohort and were therefore excluded from the bivariate analysis. The bivariate random-effects model yielded a pooled sensitivity of 0.666 (95% CI: 0.578–0.744) and a pooled specificity of 0.919 (95% CI: 0.842–0.960). The corresponding SROC curve is shown in Figure 3. Spearman rank correlation analysis showed a significant positive correlation between sensitivity and the false-positive rate (*ρ* = 0.790, *p* = 0.020), suggesting a threshold effect; however, this analysis was based on only eight studies and should therefore be interpreted cautiously (Supplementary Figure S1).

**Figure 2:**
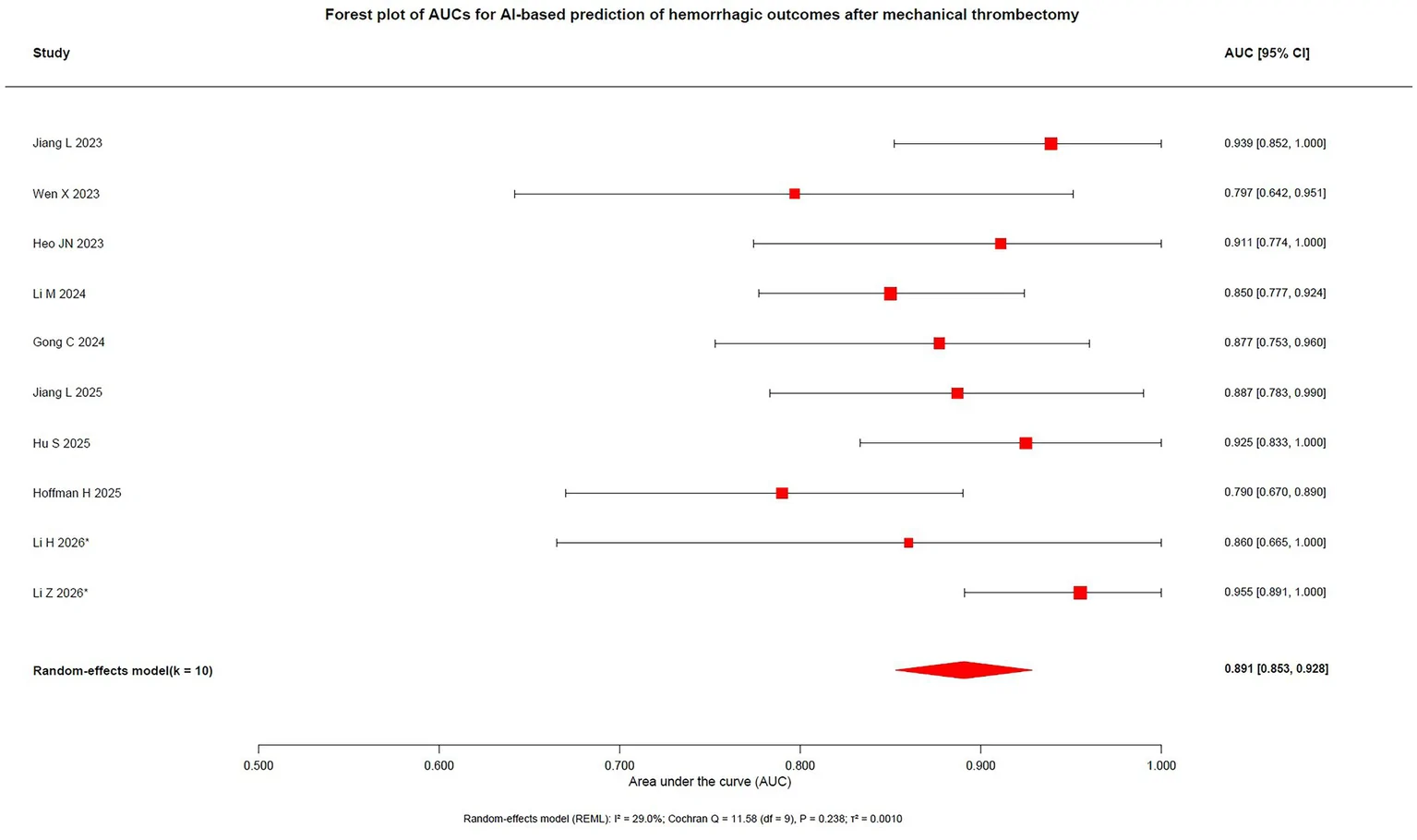
Forest Plot of the pooled AUC for AI-driven imaging-based prediction models.

**Figure 3:**
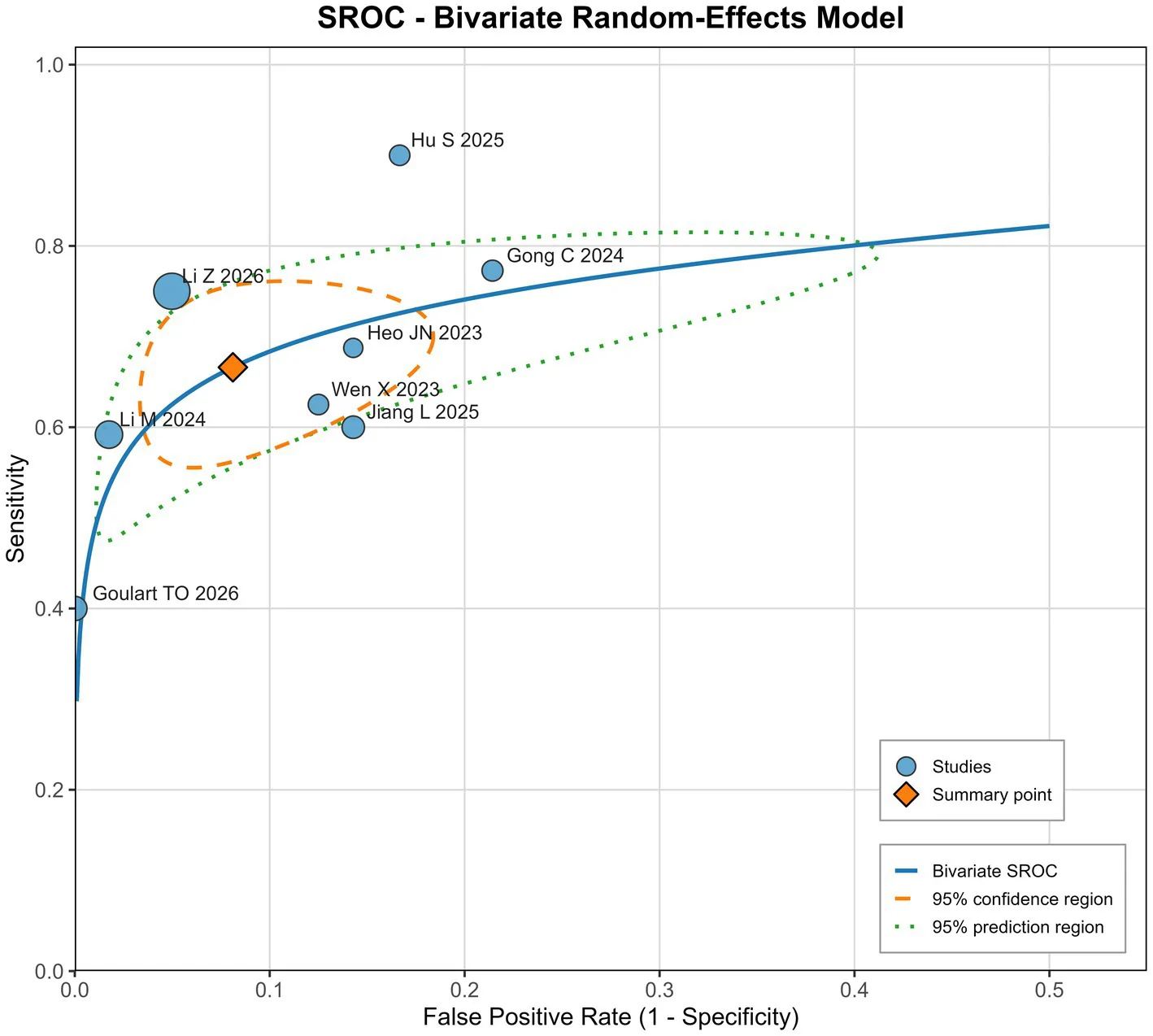
SROC curve based on the bivariate random-effects model.

#### 3.2.2 Subgroup analyses

To explore potential sources of heterogeneity, five exploratory subgroup analyses were performed (Table 2, Supplementary Figure S2).

**Table 2:** Overall meta-analysis and subgroup analysis results.

Analysis	Subgroup	No. of studies	Pooled AUC (95% CI)	I^2^ (%)	P for heterogeneity	P for subgroup difference
Overall	—	10	0.891 (0.853–0.928)	29.0	0.238	—
Imaging timing	Pre-procedural	5	0.861 (0.808–0.914)	29.0	0.238	0.048
	Post-procedural	5	0.927 (0.884–0.971)	0	0.769	
Predictor type	Image + clinical	6	0.907 (0.858–0.956)	34.9	0.194	0.151
	Image-only	4	0.860 (0.808–0.911)	0	0.718	
Validation type	External	3	0.915 (0.849–0.981)	57.8	0.091	0.236
	Internal	7	0.872 (0.828–0.916)	0	0.579	
Outcome definition	HT	6	0.902 (0.859–0.945)	0	0.695	0.878
	ICH	1	0.850 (0.777–0.924)	—	—	
	sICH	2	0.879 (0.718–1.000)*	84.5	0.011	
	Broad HT	1	0.860 (0.665–1.000)*	—	—	
Country	China	8	0.901 (0.863–0.939)	21.2	0.356	0.239
	Non-China	2	0.843 (0.726–0.961)	45.1	0.177	

\UC, area under the curve; CI, confidence interval; HT, hemorrhagic transformation; ICH, intracranial hemorrhage; sICH, symptomatic intracranial hemorrhage. *The calculated upper limit of the 95% CI exceeded 1.000 and was therefore truncated at 1.000.

A nominal between-subgroup difference was observed according to imaging timing (P for subgroup difference = 0.048). Given the exploratory nature of the subgroup analyses and the absence of multiplicity adjustment, this finding should be interpreted cautiously. The pooled AUC was 0.861 (95% CI: 0.808–0.914) for pre-procedural imaging models and 0.927 (95% CI: 0.884–0.971) for post-procedural imaging models. When stratified by predictor type, the pooled AUC was 0.860 (95% CI: 0.808–0.911) for image-only models and 0.907 (95% CI: 0.858–0.956) for image-plus-clinical models, with no significant subgroup difference (*p* = 0.151). According to validation type, the pooled AUC was 0.915 (95% CI: 0.849–0.981) for externally validated models and 0.872 (95% CI: 0.828–0.916) for internally validated models, with no significant subgroup difference (*p* = 0.236). No significant subgroup difference was observed according to outcome definition (*p* = 0.878) or country (*p* = 0.239). Detailed results of the subgroup analyses are presented in Table 2.

#### 3.2.3 Sensitivity analysis

Leave-one-out sensitivity analysis of the pooled AUC showed that the summary estimate remained stable after sequential exclusion of individual studies, with pooled AUCs ranging from 0.878 to 0.902, compared with the primary pooled AUC of 0.891 (95% CI: 0.853–0.928). These findings indicated that no single study materially influenced the overall pooled AUC estimate. In particular, exclusion of Li et al. (31), which reported mean performance across repeated random train-test splits, yielded a pooled AUC of 0.891 (95% CI: 0.852–0.930), essentially unchanged from the primary estimate (Supplementary Figure S3).

Leave-one-out sensitivity analysis of the bivariate random-effects model also showed that the pooled sensitivity and specificity remained broadly stable after sequential exclusion of each eligible study, indicating that no single study had a substantial influence on the summary diagnostic performance estimates (Supplementary Figure S4).

#### 3.2.4 Calibration and clinical utility

Calibration information was reported in 5 of the 11 included studies, whereas decision-curve analysis was reported in 5 studies. The reported approaches and metrics varied substantially across studies; therefore, these findings were summarized descriptively rather than quantitatively pooled. For Goulart et al. (34), the reported calibration assessment pertained to mSVD-based logistic regression models rather than the XGBoost model used for sensitivity/specificity extraction. Detailed calibration and decision-curve information is presented in Supplementary Table S1.

### 3.3 Quality assessment results

The risk of bias of the included studies was assessed using the PROBAST+AI tool, with results summarized in Figure 4 and Supplementary Table S2. In the Participants and Data Sources domain, nine studies were rated as low risk and two as high risk. All eleven studies were rated as low risk in the Predictors domain. In the Outcome domain, eight studies were rated as low risk and three as high risk. The Analysis domain represented the major source of risk of bias: nine studies were rated as high risk, one as unclear risk, and only one as low risk. The main concerns included limited numbers of outcome events relative to model complexity, model selection or comparison using evaluation datasets, small validation samples, and incomplete assessment or reporting of calibration and uncertainty. Overall, ten studies were rated as having a high risk of bias and one as having an unclear risk of bias.

**Figure 4:**
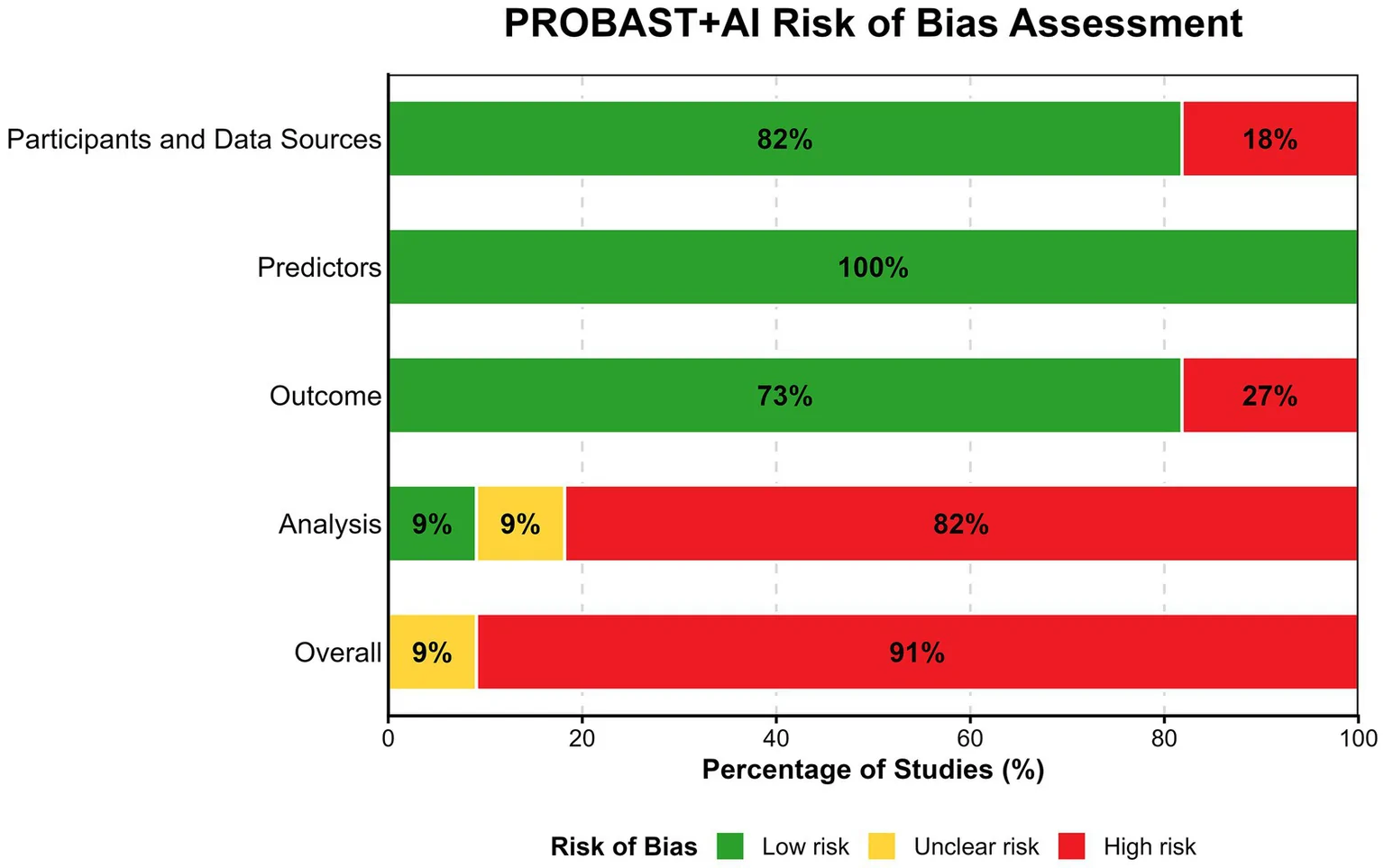
PROBAST+AI risk of bias assessment.

## 4 Discussion

### 4.1 Main findings

This systematic review and meta-analysis included 11 studies involving 3,541 patients and evaluated the performance of AI-driven imaging-based models for predicting hemorrhagic transformation after mechanical thrombectomy. Ten studies contributed eligible AUC data, yielding a pooled AUC of 0.891 (95% CI: 0.853–0.928), with low between-study heterogeneity (*I*^2^ = 29.0%). In the bivariate analysis of eight studies, the pooled sensitivity was 0.666 (95% CI: 0.578–0.744) and the pooled specificity was 0.919 (95% CI: 0.842–0.960), with higher pooled specificity than sensitivity. Exploratory subgroup analyses showed a nominal difference according to imaging timing, whereas no statistically significant subgroup differences were observed according to predictor type, validation type, outcome definition, or country. However, these subgroup findings should be interpreted cautiously and regarded as hypothesis-generating. However, the predominance of retrospective studies and the high overall risk of bias, particularly in the analysis domain, indicate that these findings should be interpreted cautiously. Importantly, the present review broadens the existing evidence base by systematically incorporating peri−/post-procedural imaging models and studies from non-China cohorts, thereby extending previous evidence beyond predominantly pre-procedural and geographically concentrated settings.

Our findings are broadly consistent with previous systematic reviews reporting favorable discrimination of imaging-based AI models for hemorrhagic transformation prediction. Salimi et al. reported pooled AUCs of 0.91 for deep learning models and 0.86 for radiomics-based models, while Wang et al. reported pooled AUCs of 0.857 for image-only models and 0.899 for image-plus-clinical models. In the present study, the pooled AUCs for image-only and image-plus-clinical models were 0.860 and 0.907, respectively. However, the difference between these two subgroups was not statistically significant (*p* = 0.151), and therefore the current evidence does not establish a clear advantage of adding clinical variables to imaging-based models.

### 4.2 Comparison with previous studies

The pooled AUC of AI-driven imaging-based prediction models in the present study was 0.891 (95% CI: 0.853–0.928), indicating generally favorable discriminative performance. However, comparisons of pooled AUCs with performance estimates reported for conventional prediction approaches across different studies should be interpreted cautiously because of differences in study populations, outcome definitions, predictor sets, and validation strategies. Therefore, such indirect comparisons cannot establish the superiority of AI-based models over conventional approaches.

Direct head-to-head comparisons within the included studies yielded mixed results. In Hoffman et al., the XGBoost model achieved a higher AUC than logistic regression (0.79 vs. 0.54), TAG (0.79 vs. 0.69), and STBA (0.79 vs. 0.45), with statistically significant differences. In Li Z et al., the TabPFN model outperformed the ASIAN and TAG scores in the external validation cohort (AUC 0.955 vs. 0.763 and 0.688, respectively), whereas its advantage over logistic regression was not statistically significant (0.955 vs. 0.949, *p* = 0.055). Hu et al. also reported no statistically significant differences in DeLong comparisons between the evaluated models. Taken together, the available evidence does not demonstrate a consistent superiority of AI-based models over conventional statistical models, although some AI models showed better discrimination than individual clinical risk scores in specific cohorts.

Previous systematic reviews have also evaluated imaging-based prediction of hemorrhagic transformation. Compared with the meta-analysis by Suh et al. (35), which reported an AUC of 0.85 for MRI-based HT prediction, our study showed a numerically higher pooled AUC. This difference, however, should be interpreted cautiously because of differences in patient populations, imaging modalities, outcome definitions, and validation strategies across studies. Furthermore, compared with the external validation study by van der Ende et al. (9), which reported AUCs of 0.51–0.61 for traditional risk scores in predicting sICH, our pooled AUC was substantially higher. However, these indirect comparisons do not establish the superiority of AI-based models over conventional approaches, and direct comparative studies with rigorous external validation are still needed.

### 4.3 Clinical implications of subgroup analyses

The subgroup analyses yielded several clinically relevant insights.

The image-plus-clinical models showed a numerically higher pooled AUC than image-only models (0.907 vs. 0.860), suggesting that clinical variables may provide complementary information to imaging features and that combined modeling may be a promising direction for future model optimization. However, the between-subgroup difference was not statistically significant (*p* = 0.151), and this finding therefore warrants further validation in future studies.

From a model validation perspective, externally validated models showed a slightly higher pooled AUC than internally validated models (0.915 vs. 0.872). However, the between-subgroup difference was not statistically significant (*p* = 0.236), and this result does not establish that externally validated models have superior predictive performance. Only three studies reported external validation results, and the validation cohorts differed across studies in terms of source population, sample size, and outcome definitions. Therefore, more multicenter, independent external validation studies are needed to rigorously evaluate the generalizability of these models.

Imaging timing showed a nominal between-subgroup difference, with a higher pooled AUC for post-procedural than for pre-procedural imaging models (0.927 vs. 0.861; P for subgroup difference = 0.048). However, this finding should be regarded as exploratory and hypothesis-generating rather than as evidence of superior predictive performance of post-procedural imaging. A major concern is potential predictor–outcome circularity or temporal overlap in several post-procedural studies. Because imaging predictors were obtained after thrombectomy and close to the assessment of hemorrhagic outcomes, some imaging features may already reflect early hemorrhagic changes, contrast extravasation, or related post-procedural abnormalities. Thus, the apparently higher discrimination of post-procedural models may partly reflect early detection of an evolving hemorrhagic process rather than prediction of a genuinely future hemorrhagic event. A biological explanation is also plausible, as post-procedural imaging may capture reperfusion-related changes, blood–brain barrier disruption, contrast leakage, and other abnormalities temporally closer to subsequent hemorrhagic transformation. Nevertheless, the imaging-timing comparison was based on a small number of studies, was sensitive to analytic assumptions, and represented one of five subgroup comparisons performed without multiplicity adjustment. Therefore, this finding should be considered hypothesis-generating and requires confirmation in future prospective studies with clearly separated predictor-acquisition and outcome-assessment windows. No statistically significant subgroup differences were observed according to outcome definition (*p* = 0.878) or country (*p* = 0.239). These findings suggest that the observed predictive performance was not clearly explained by these factors in the current evidence base. However, the small number of studies within several subgroups and the methodological heterogeneity across studies limit the strength of these comparisons, and the results should therefore be regarded as exploratory. Although the single MRI-based study reported relatively favorable discrimination, this may partly reflect the richer tissue characterization and multiparametric information provided by MRI. However, this observation was derived from only one study and should not be interpreted as evidence that MRI is superior to CT- or DSA-based approaches.

### 4.4 Clinical implications

The findings of this study suggest that AI-driven imaging-based models may have potential clinical value in assisting HT risk assessment. With a pooled AUC of 0.891 (95% CI: 0.853–0.928), these models may help identify patients at increased risk of hemorrhagic complications after MT and provide additional information for postoperative risk stratification and individualized monitoring. For models based on pre-procedural imaging, the results may further offer risk information before or during treatment planning, potentially supporting more individualized periprocedural management. However, discrimination alone does not establish clinical utility, and the current evidence is insufficient to support direct changes in treatment decisions, such as blood pressure targets or antithrombotic therapy, based on these models.

The exploratory subgroup analyses in this study offer potential directions for future model development. Image-plus-clinical models showed a numerically higher pooled AUC than image-only models (0.907 vs. 0.860), although the between-subgroup difference was not statistically significant (*p* = 0.151). The imaging-timing subgroup finding should be regarded as hypothesis-generating because of potential predictor–outcome overlap and statistical uncertainty, and the current evidence does not establish superiority of post-procedural over pre-procedural imaging. Nevertheless, these subgroup findings should be regarded as exploratory because of the small number of studies and differences in patient populations, imaging protocols, outcome definitions, and model development strategies.

The included studies used a range of imaging modalities, including CT, DSA, and MRI, suggesting that AI-based prediction may be applicable to different imaging settings. However, the current evidence does not permit reliable comparisons of predictive performance between individual imaging modalities. Clinical translation will therefore require standardized imaging and modeling workflows, clearly defined outcome criteria, and prospective evaluation in representative patient populations.

Furthermore, the lack of independent external validation in most studies highlights a critical research gap. Calibration and decision-curve analyses were also inconsistently reported, limiting assessment of whether the reported discrimination would translate into clinically meaningful benefit. High-quality multicenter external validation, together with comprehensive assessment of calibration and clinical utility, is therefore a key step in advancing these AI models from model development toward clinical application.

### 4.5 Strengths

This study has several notable strengths. First, it specifically focused on AI-driven imaging-based models for predicting HT after MT, thereby providing a focused synthesis of this clinically relevant evidence base. Second, the search strategy was comprehensive, covering eight Chinese and English databases including PubMed, Embase, Cochrane Library, Web of Science, CNKI, Wanfang, VIP, and CBM, which helped broaden literature coverage and reduce the risk of language-related omission. Third, the inclusion criteria were rigorous, requiring eligible studies to evaluate AI-driven prediction models incorporating imaging or imaging-derived predictors in patients treated with MT, thereby ensuring consistency with the final eligibility framework. Fourth, the subgroup analyses systematically explored potential sources of heterogeneity across five dimensions—imaging timing, predictor type, validation type, outcome definition, and country—which enriched the depth of interpretation. Fifth, the study protocol was prospectively registered with PROSPERO (CRD420261441463), and deviations from the registered protocol are explicitly reported in the Methods.

### 4.6 Limitations

This study has several limitations. First, most included studies were retrospective in design, which may have introduced selection bias and limited the strength of causal or prospective inference. Second, the definitions and assessment criteria for HT varied across studies, which may have increased clinical heterogeneity. Third, some studies did not report the 95% CI of the AUC, and these values had to be estimated when sufficient information was available; such estimation may have introduced some degree of uncertainty. In Li H 2026, performance was reported as an average across repeated random train-test splits rather than from a single fixed evaluation cohort, which adds further uncertainty to the study-level precision estimate; however, excluding this study in leave-one-out analysis did not materially change the pooled AUC. In addition, not all studies reported paired sensitivity and specificity data suitable for the bivariate SROC analysis, which limited the number of eligible studies. A significant threshold effect was also observed in the bivariate analysis, although this finding should be interpreted cautiously given the limited number of eligible studies. Fourth, most studies lacked independent external validation, with only three studies reporting external validation, so the generalizability of the models requires further investigation. Fifth, variations in imaging acquisition and AI modeling workflows across studies may have affected the comparability of the results. In addition, the small number of studies within several subgroups limited the robustness of subgroup comparisons, and the observed difference according to imaging timing should therefore be interpreted as exploratory, particularly because multiple subgroup comparisons were examined without multiplicity adjustment. Potential predictor–outcome circularity in several post-procedural studies may also have inflated the apparent discrimination of these models and further limits interpretation of the imaging-timing subgroup finding. Sixth, the overall risk of bias was high in most included studies, particularly in the analysis domain, owing to issues such as limited numbers of outcome events relative to model complexity, small validation samples, and potential optimism arising from model selection and evaluation procedures. Calibration and decision-curve analyses were also inconsistently reported, limiting assessment of the clinical applicability of the models. Finally, variability in imaging protocols across studies precluded more detailed quantitative subgroup analyses by specific MRI sequences or CT parameters, and future studies with standardized imaging protocols are needed to enable such comparisons.

## 5 Conclusion

A key contribution of this systematic review is the inclusion of both pre- and post-procedural imaging models and evidence from China and non-China cohorts, providing a broader view of AI-driven imaging-based prediction of HT after MT. Overall, the included models showed generally favorable reported discrimination, with a pooled AUC of 0.891 (95% CI: 0.853–0.928). Exploratory subgroup analyses did not provide robust evidence supporting the superiority of any specific subgroup and should be interpreted cautiously because of the limited number of studies and methodological heterogeneity. However, these subgroup findings should be interpreted cautiously because of the limited number of studies and methodological heterogeneity. The current evidence is predominantly derived from retrospective studies, with limited independent external validation and a high overall risk of bias, particularly in the analysis domain. Therefore, the generalizability and clinical applicability of these models remain uncertain. Future large-scale, multicenter, prospective studies with rigorous external validation, calibration assessment, and evaluation of clinical utility are needed before these models can be translated into routine clinical practice.
