# Supplementary material for: Artificial intelligence-driven imaging-based prediction models for hemorrhagic transformation after mechanical thrombectomy in acute ischemic stroke: a systematic review and meta-analysis
Source: Front Neurol. 2026 Oct 20;17:1960029. doi: 10.3389/fneur.2026.1960029 (PMC13727432; doi:10.3389/fneur.2026.1960029)
Supplement: Supplementary file 1 [file Supplementary_file_1.docx]

Search period: from database inception to July 2, 2026.

Wanfang search strategy:

卒中 OR 脑卒中 OR 脑梗死 OR 急性脑缺血

AND

机械取栓 OR 机械血栓切除术 OR MT OR 取栓

AND

人工智能 OR 机器学习 OR 深度学习 OR 深度学习 OR "AI" OR "ML" OR "DL" OR 影像组学 OR "Radiomics"

AND

出血转化 OR "出血性转化" OR HT OR 出血性转变

CNKI search strategy:

急性缺血性脑卒中 + 急性缺血性脑卒中病人 + 急性缺血性脑卒中溶栓 + 急性缺血性脑卒中偏瘫 + 急性缺血性脑卒中复发 + 急性缺血性脑卒中疾病

AND

出血转化 + '出血转化(ht)' + 出血转化风险

AND

机械取栓 + 机械取栓治疗 + 机械取栓术 + 机械取栓装置 + 机械取栓技术 + 机械取栓绿色通道

VIP search strategy:

(急性缺血性脑卒中 OR 急性缺血性卒中 OR 缺血性脑卒中 OR 缺血性卒中 OR 急性脑梗死 OR 脑梗死)

AND

(机械取栓 OR 血管内取栓 OR 血管内治疗 OR 动脉取栓 OR 支架取栓)

AND

(出血转化 OR 出血性转化 OR 颅内出血 OR 症状性颅内出血 OR 脑出血)

AND

(人工智能 OR 机器学习 OR 深度学习 OR 神经网络 OR 预测模型 OR 列线图 OR 影像组学 OR 放射组学)

CBM/SinoMed search strategy:

急性缺血性脑卒中 OR 急性缺血性卒中 OR 缺血性脑卒中 OR 缺血性卒中 OR 急性脑梗死 OR 脑梗死 OR 脑梗塞

机械取栓 OR 血管内取栓 OR 血管内治疗 OR 动脉取栓 OR 支架取栓 OR 血栓切除术

出血转化 OR 出血性转化 OR 颅内出血 OR 症状性颅内出血 OR 脑出血 OR 出血并发症

人工智能 OR 机器学习 OR 深度学习 OR 神经网络 OR 卷积神经网络 OR 预测模型 OR 列线图 OR 影像组学 OR 放射组学

PubMed：

(

"Stroke"[Mesh] OR "Brain Ischemia"[Mesh] OR "Cerebral Infarction"[Mesh]

OR "acute ischemic stroke"[tiab] OR "acute ischaemic stroke"[tiab]

OR "ischemic stroke"[tiab] OR "ischaemic stroke"[tiab]

OR AIS[tiab] OR "large vessel occlusion"[tiab] OR LVO[tiab]

)

AND

(

"Thrombectomy"[Mesh] OR "Endovascular Procedures"[Mesh]

OR thrombectom*[tiab]

OR "mechanical thrombectomy"[tiab]

OR "endovascular thrombectomy"[tiab]

OR "endovascular treatment"[tiab]

OR "endovascular therapy"[tiab]

OR EVT[tiab]

)

AND

(

"Artificial Intelligence"[Mesh] OR "Machine Learning"[Mesh]

OR "Deep Learning"[Mesh] OR "Radiomics"[Mesh]

OR "artificial intelligence"[tiab] OR AI[tiab]

OR "machine learning"[tiab] OR ML[tiab]

OR "deep learning"[tiab] OR DL[tiab]

OR radiomic*[tiab]

OR "texture analysis"[tiab]

OR "neural network*"[tiab]

OR CNN[tiab]

OR "support vector machine"[tiab]

OR SVM[tiab]

OR "random forest"[tiab]

OR XGBoost[tiab]

)

AND

(

"hemorrhagic transformation"[tiab]

OR "haemorrhagic transformation"[tiab]

OR "intracranial hemorrhage"[tiab]

OR "intracranial haemorrhage"[tiab]

OR "intracerebral hemorrhage"[tiab]

OR "intracerebral haemorrhage"[tiab]

OR "symptomatic intracranial hemorrhage"[tiab]

OR "symptomatic intracerebral hemorrhage"[tiab]

OR sICH[tiab]

OR "parenchymal hematoma"[tiab]

OR "parenchymal haematoma"[tiab]

)

AND

(

predict*[tiab]

OR prognos*[tiab]

OR model*[tiab]

OR nomogram[tiab]

OR classifier*[tiab]

OR classification[tiab]

OR "risk assessment"[tiab]

OR "risk prediction"[tiab]

OR validation[tiab]

)

WOS：

TS=("stroke" OR "brain ischemia" OR "brain ischaemia" OR "cerebral infarction" OR "acute ischemic stroke" OR "acute ischaemic stroke" OR "ischemic stroke" OR "ischaemic stroke" OR AIS OR "large vessel occlusion" OR LVO) AND TS=(thrombectom* OR "mechanical thrombectomy" OR "endovascular thrombectomy" OR "endovascular treatment" OR "endovascular therapy" OR EVT) AND TS=("artificial intelligence" OR AI OR "machine learning" OR ML OR "deep learning" OR DL OR radiomic* OR "texture analysis" OR "neural network*" OR "neural networks" OR CNN OR "convolutional neural network*" OR "support vector machine" OR SVM OR "random forest" OR XGBoost) AND TS=("hemorrhagic transformation" OR "haemorrhagic transformation" OR "intracranial hemorrhage" OR "intracranial haemorrhage" OR "intracerebral hemorrhage" OR "intracerebral haemorrhage" OR "symptomatic intracranial hemorrhage" OR "symptomatic intracerebral hemorrhage" OR "symptomatic intracranial haemorrhage" OR "symptomatic intracerebral haemorrhage" OR sICH OR "parenchymal hematoma" OR "parenchymal haematoma") AND TS=(predict* OR prognos* OR model* OR nomogram OR classifier* OR classification OR "risk assessment" OR "risk prediction" OR validation )

Embase:

('ischemic stroke'/exp OR 'brain ischemia'/exp OR 'middle cerebral artery occlusion'/exp OR 'reperfusion injury'/exp OR ('ischemi*' NEAR/3 'stroke'):ti,ab OR ('cerebrovascular' NEAR/3 'accident'):ti,ab OR ('brain' NEAR/3 'infarct*'):ti,ab OR ('cerebral' NEAR/3 'infarct*'):ti,ab OR mcao:ti,ab OR ('reperfusion' NEAR/3 'injur*'):ti,ab) AND ('thrombectomy'/exp OR 'mechanical thrombolysis'/exp OR 'endovascular surgery'/exp OR 'stent retriever'/exp OR ('mechanical' NEAR/3 'thrombectom*'):ti,ab OR ('endovascular' NEAR/3 'thrombectom*'):ti,ab OR ('stent' NEAR/3 'retriever'):ti,ab OR ('aspiration' NEAR/3 'thrombectom*'):ti,ab OR solitaire*:ti,ab OR trevo*:ti,ab OR penumbra*:ti,ab OR mt:ti,ab) AND ('hemorrhage'/exp OR 'intracranial hemorrhage'/exp OR 'cerebral hemorrhage'/exp OR 'hemorrhagic transformation'/exp OR ('hemorrhagic' NEAR/3 'transformation'):ti,ab OR ht:ti,ab OR ('intracranial' NEAR/3 'hemorrhage'):ti,ab OR ('cerebral' NEAR/3 'hemorrhage'):ti,ab OR ('parenchymal' NEAR/3 'hematoma'):ti,ab OR ph:ti,ab OR hi:ti,ab) AND ('artificial intelligence'/exp OR 'deep learning'/exp OR 'machine learning'/exp OR 'neural network'/exp OR 'radiomics'/exp OR 'nomogram'/exp OR ('artificial' NEAR/3 'intelligenc*'):ti,ab OR ('deep' NEAR/3 'learning'):ti,ab OR ('machine' NEAR/3 'learning'):ti,ab OR ('neural' NEAR/3 'network*'):ti,ab OR radiomic*:ti,ab OR nomogram*:ti,ab OR ('convolutional' NEAR/3 'neural'):ti,ab OR cnn:ti,ab OR ('random' NEAR/3 'forest*'):ti,ab OR ('support' NEAR/3 'vector'):ti,ab OR svm:ti,ab OR ('gradient' NEAR/3 'boosting'):ti,ab OR xgboost:ti,ab)

Cochrane Library：

((((stroke OR ischemia OR infarction OR cerebrovascular accident):ti,ab,kw) AND ((thrombectomy OR endovascular OR stent retriever OR aspiration):ti,ab,kw) AND ((hemorrhage OR hemorrhagic transformation OR HT OR bleeding):ti,ab,kw) AND ((artificial intelligence OR machine learning OR deep learning OR radiomics OR nomogram OR CNN OR SVM):ti,ab,kw)))
